# The Link Between Environment, Age, and Health in a Large Cohort of Companion Dogs from the Dog Aging Project

**DOI:** 10.1093/geroni/igab046.3535

**Published:** 2021-12-17

**Authors:** Brianah McCoy, Layla Brassington, Greer Dolby, Kelly Jin, Devin Collins, Matthew Dunbar, Noah Snyder-Mackler

**Affiliations:** 1 Arizona State University, Tempe, Arizona, United States; 2 Allen Institute for Brain Science, Allen Institute for Brain Science, Washington, United States; 3 University of Washington, University of Washington, Washington, United States; 4 University of Washington, UNiversity of Washington, Washington, United States

## Abstract

Exposure to social environmental adversity strongly predicts health and survival in many species such as non-human primates, wild mammals, and humans. However, little is known about how the health and mortality effects of these social determinants vary across the lifespan. Using the companion dog, which serves as a powerful comparative model for human health and aging due to our shared biology and environment, we examined which components of the social environment impact health, and how the effects vary with age, in dogs. We first drew on detailed survey data from owners of 27,547 dogs from the Dog Aging Project and identified six factors that together explained 35% of the variation in dog’s social environment. These factors all predicted measures of health, disease, and mobility, when controlling for dog age and weight. Factors capturing measures of financial and household adversity were linked to poorer companion dog health, while factors associated with the social companions, like dogs and adults, were linked to better health. Interestingly, some of these effects differed across a dog’s lifespan: for instance, the effect of neighborhood disadvantage on disease instances was strongest in older dogs. Together, our findings point to similar links between adversity and health in companion dogs, and set up future work on the molecular and biological changes associated with environmental variation in order to identify ways to mitigate or even reverse the negative environmental effects.

